# LoRaWAN Performance Analysis for a Water Monitoring and Leakage Detection System in a Housing Complex

**DOI:** 10.3390/s22197188

**Published:** 2022-09-22

**Authors:** Atheer M. Alghamdi, Enas F. Khairullah, Mohammad M. Al mojamed

**Affiliations:** 1 Information Technology Department, Faculty of Computers and Information Technology, King Abdulaziz University, Jeddah 23713, Saudi Arabia; 2 Computer Science Department, Computing College-Al-Qunfudah, UMM Al-QURA University, Al-Qunfudah 28814, Saudi Arabia

**Keywords:** LoRaWAN, LPWAN, IoT, FloRa, water monitoring, leakage detection, housing complex

## Abstract

The automation of water leakage detection and monitoring systems has recently been made possible by the Internet of Things (IoT). However, the high cost is an obstacle when applying a network over a large area. The Low-Power Wide-Area Network (LPWAN) was created specifically to address long-range IoT applications. The Long-Range Wide-Area Network (LoRaWAN) is one of the most common LPWANs. In this study, a method for monitoring and detecting water leakage in a housing complex was tested using LoRaWAN. Water leakage was detected using a low-pressure system model comprising a water meter, presser sensor, and smart valve within a LoRa node. This study investigates the use of LoRaWAN for water monitoring and leakage detection by implementing a comprehensive case study to identify LoRaWAN’s feasibility, reliability, and scalability for water monitoring and leakage detection in simulated scenarios. The housing complex varied in size and number of nodes. The LoRaWAN was evaluated by the FloRa simulator package through the Objective Modular Network Testbed (OMNeT++) platform. The results indicated that it was an efficient means of water monitoring and leakage detection in housing complexes.

## 1. Introduction

Water distribution systems that span kilometers require a long-range network to monitor and detect water leakage. Many systems use a Wi-Fi or cellular network, but these are not feasible in large network areas, such as a housing complex, because of the high cost.

In view of the continuing expansion of the Internet of Things (IoT), a Low-Power Wide-Area Network (LPWAN) would be an ideal solution because it covers a wide area where the data rate is low [1]. There are three common LPWANs: Narrowband Internet of Things (NB-IoT), Sigfox, and Long-Range Wide-Area Network (LoRaWAN) [2]. LoRaWAN is considered the most popular because of its advantages in bandwidth scalability, low power, high robustness, wide range coverage, localization, and overall improved network capacity [3,4].

LoRaWAN uses LoRa technology, which was introduced by SemTech and later developed by the LoRa Alliance [5]. LoRaWAN architecture is a star topology, which means that the end devices communicate via LoRaWAN gateways, not directly with each other [6].

The wireless LoRaWAN communication protocol was built by LoRa Alliance to address long-range communication in the IoT by reducing costs and eliminating repeaters to decrease network complexity [7]. In other words, it is a media access control (MAC) protocol that supports sensor networks [8]. In addition, LoRaWAN security has demonstrated high confidentiality, integrity, and availability since its release [9]. Typically, LoRaWAN has three main components: end nodes, gateways, and a network server. It is applicable where high energy is not required to transfer data from sensors, such as in fire detection and smart cities, farms, and healthcare [10]. The range of LoRaWAN is 2–5 km in urban, 15 km in suburban, and 45 km in rural areas [3].

LoRaWAN includes three classes of end devices, each of which has specific characteristics. A brief description of classes A, B, and C is given in [11]. Class A schedules the uplink based on needs only; Class B schedules slots to receive periodic messages; and Class C remains open to receive messages at any time. Class A consumes little energy and supports all devices. Class B consumes more energy, and Class C, which listens continuously with low latency, consumes the most.

Activation By Personalization (ABP) and Over-The-Air Activation (OTAA) are two techniques for activating a LoRaWAN network [12]. ABP is used when the device is ready to connect via the network without any additional procedure, and OTAA is used to send a join request from the end device to the application server. Moreover, Adaptive Data Rate (ADR) is the most used mechanism for LoRaWAN parameter management. ADR is developed to effectively configure the data rate and transmitted power of a number of nodes in order to maximize the system capacity [13]. The main parameters of LoRaWAN are Spreading Factors (SFs), BandWidth (BW), Center Frequency (CF), Transmission Power (TP), and Coding Rate (CR) [14].

Water leakage causes the loss of 2000–20,000 gallons of water per year for each home. While some leakages are visible, others are not, and they damage both the house and the housing complex. Therefore, the homeowner and the housing complex operator need a LoRaWAN network to monitor and reduce water leakage as much as possible. Some water monitoring and leakage detection systems use Wi-Fi, cellular, and Bluetooth networks, but these are not feasible because of the high cost in a large area [15]. Therefore, this study considers LoRaWAN as the most efficient and cost-effective monitoring system for housing complexes.

This research focuses on investigating the feasibility of LoRaWAN for water monitoring and leakage detection in a housing complex. The system model used the FLoRa framework on the OMNeT++ platform. Several scenarios with and without water leakage were discussed. A LoRa node monitored and detected water leakage through three devices: a water meter sensor to measure water consumption every half hour, a water pressure sensor to measure water pressure if it falls below 2.7 kPa based on [15] and send an updated message to the network every five minutes, and a smart valve sensor to control the water flow into the house by turning it on and off. The LoRaWAN was simulated in FloRa [13] for evaluation.

The key contributions of the study are to develop a realistic network model to simulate water monitoring and leakage detection for housing complexes and to comprehensively investigate LoRaWAN’s feasibility for such purposes, where the model was evaluated in scenarios consisting of up to 1800 nodes spreading over 25 km².

The remainder of this paper is structured as follows: Section 2 gives the background of LoRaWAN technology and reviews the related studies of LoRaWAN-based water system applications. Section 3 explains the proposed system of this research. Additionally, Section 4 presents a discussion of the results and summarizes the most valuable information. Finally, the conclusions and future work are presented in Section 5 to conclude the research.

## 2. Background

### 2.1. Long-Range Wide-Area Network (LoRaWAN)

This section presents LoRa technology and LoRaWAN network, which are used in long-distance and low-data rate applications. Also, the main concepts of the LoRaWAN network are indicated and described.

#### LoRa/LoRaWAN

LoRa is an unlicensed radio frequency in the Industrial, Scientific, and Medical (ISM) band used for low-power, long-distance wireless communication. As a physical layer, it is used for long-distance data transfer, decreasing repeaters, reducing device prices, increasing battery life and overall network capacity, and accommodating a variety of devices [16].

To address issues with long-range communication in the IoT, the LoRa Alliance created the LoRaWAN wireless communication protocol. Battery life, dependability, security, and the range of applications that can be supported are all significantly affected by the LoRaWAN architecture [7].

**LoRaWAN Architecture**

**Figure 1:**
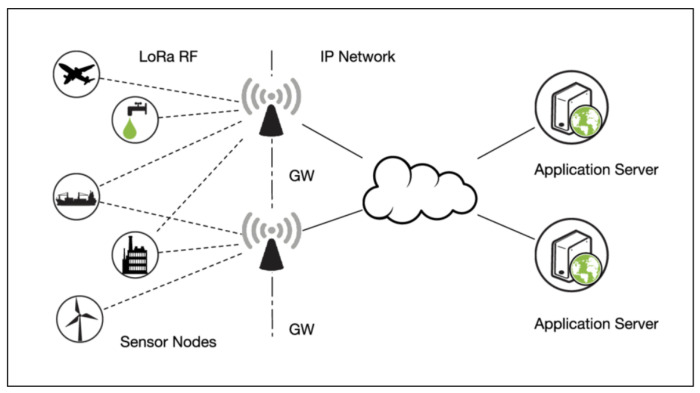
General architecture of LoRaWAN [18].

To use LoRaWAN, end devices must communicate with gateways. Using a quicker backhaul connection, gateways transmit LoRaWAN packets from the nodes to a network server. Because it considers the packets that must be returned to the nodes, gateways are bidirectional switches [8]. The architecture of LoRaWAN is illustrated in Figure 1 and is composed of the following three basic elements [5,17].

**LoRaWAN End Devices**

A LoRaWAN end device is a low-energy sensor that connects to gateways via LoRaWAN radio. The end devices are called LoRaWAN nodes or endpoints, such as sensors for humidity, pressure, temperature, and position.

**LoRaWAN Gateways**

A LoRaWAN gateway is an intermediate device that forwards data from LoRa nodes to a network server across an Internet protocol data channel, such as Ethernet or cellular. A LoRaWAN setup can have more than a gateway, and the same datagram can be handled by different gateways. It uses regular Internet Protocol (IP) communication to join the network server and acts as a channel for incoming network connection frames but does not interpret the contents [10].

**LoRaWAN Network Server**

A LoRaWAN network server can analyze and create packets to send back to devices. It is considered the core of the whole system (radio resource administration, access control, and protection). The LoRaWAN network server performs packet inspection and immediately discards those that are invalid, thereby enhancing security by reducing computer use. It also supports a large number of services: duplicated packet filtering, network management technique execution, acknowledgment management, and sending application-level data to another server are examples [10].

### 2.2. Related Work

Water monitoring and leakage detection in water distribution systems have been the topic of extensive and varied research in recent years. In the Global System for Mobile Communications (GSM), the authors in [19] proposed a system to monitor water quality in terms of turbidity and temperature. Sensors were placed in pipes to measure water quality, and the GSM module transmitted the data to an Android application through the cloud. The results were updated to show temperature and water quality. Intrusion into sensor data communication across wireless networks, though, must be avoided. In [20], researchers developed an Android application with an interface to monitor a home dual-tank system using LoRaWAN. It consisted of a water pump, ultrasonic sensor, and several devices to allow the user to monitor the water quality. The system monitored the quality of the water in the upper tank for turbidity and potential of hydrogen (pH). The application requested the water level of the tank and controlled the pump operation. Furthermore, it remotely controlled the valve by turning it on and off. However, the ultrasonic sensory reliability was not sufficient. The authors in [20] used communication, and those in [21] used cellular communication. Their proposed system depended on integrating the IoT with sensors that also collected data on rain and water temperature. The result was a successful interaction between the web application and the sensor, but the system could be more effective if it were used in a wider range of scenarios.

Low-power wireless networks have emerged as a solution to the problems of long-range applications such as water distribution. To detect water leakage, the authors in [22] assessed LoRaWAN, NB-IoT, and Sigfox for transmission time and energy consumption. The NB-IoT was practical in data transmission but not in energy consumption. On the other hand, Sigfox and LoRaWAN were the best in energy consumption, but the LoRaWAN was better for data transmission. The authors in [23] discussed the challenges faced by short-range networks that have high energy consumption costs. They explained that an LPWAN provided the best water quality monitoring. In this comparison, they stated that a LoRaWAN network was suitable for urban and rural areas because it did not require a line of sight.

A novel analytical system was proposed by [24] using LoRaWAN with appropriate algorithms for smart water networks, and as a result, data compression helped in reducing storage space and power usage. However, the data limit needed to be raised to become more sensitive to anomalies. Likewise, the researchers in [25] proposed the watergrid-sense technique to monitor a smart water system through the LoRaWAN network. It was used in water networks and addressed the problem of harsh environments. The authors uploaded a large quantity of data to the server, showing that the communication system was not impervious to tough conditions within harsh environments.

The authors in [26] proposed a water meter in an experimental laboratory for smart water consumption measurement and leakage detection using LoRaWAN. The water leak was assumed when the water flow was less than 100 L. The implementation was a perfect solution, but it still needs a better network connection. Furthermore, the authors in [27] used LoRaWAN to stop water theft by enabling administrators to monitor the water systems. The operating cost was considerably low because the consumers did not have to pay for data transmission between the water system end devices and the centralizer.

The authors in [28] proposed a Smart Water Metering System (SWATS)—a mechanical water meter with a sensor and reader—to monitor consumption. Any reading that surpassed a predetermined threshold represented an abnormal situation. The technique was adequate, but it was not tested in cold environments. Additionally, the system proposed in [29] used LoRaWAN to detect leakages inside pipes in large buildings. The results of this study contributed to reducing the time and effort in discovering and eliminating water leakages. Additionally, the authors in [30] proposed a dynamic water management solution using a LoRaWAN to manage distribution and control usage. The proposed model was effective for monitoring and detecting leakages, but the authors of this study did not implement this model.

To determine how many smart water meters can connect to a gateway, the authors in [31] built a simulated system using Network Simulator 3 (NS3). Water meters were installed in buildings to track water consumption; hence, the equipment is static. According to research in Tunisia, the main findings show that a single gateway may house thousands of nodes. Due to low costs, LoRaWAN is suitable for smart water metering.

The authors in [32] used LoRaWAN technology in the Advanced Metering Infrastructure (AMI). Water is one of the resources measured in the AMI. Water consumption data are collected by the AMI and then sent to the server. The authors used the Forsk Atoll simulator to calculate the scalability of LoRaWAN in rural, suburban, and urban areas. The simulation was applied to three cities in Indonesia. The results concluded on the number of LoRaWAN gateways needed to cover the three cities: specifically, two for Padang Sidempuan city at 114.66 km${}^{2}$; four gateways for Pematang Siantar city at 55.66 km${}^{2}$; and sixteen gateways for Medan city at more than 256 km${}^{2}$. The difference in the number of gateways is due to the area of the cities. As the coverage area increases, more gateways are needed.

In [33], the researchers assessed the effectiveness of three LoRaWAN frequencies in a water quality monitoring application using MATLAB. The investigation used the frequencies 433, 865, and 915 MHz. The simulation was used to communicate water quality characteristics such as temperature, pH, and turbidity. According to the findings of the study, the transmission frequency of 433 MHz was suitable for extended battery life scenarios for its relatively low power consumption, and 868 MHz was the practical choice for a moderate transmitter with LoRaWAN applications. The power consumption of applications running at 915 MHz was higher, although data packet losses were minimal. The allowed SF value was influenced by the distance between the gateway and the LoRaWAN node (e.g., 7 for an end device close to the LoRaWAN gateway; 12 if far away).

LoRaWAN-based simulated applications for water management systems are summarized in Table 1. The study of [31] focused on LoRaWAN scalability in the water metering system. Furthermore, there are three simulated cities to measure LoRaWAN scalability for the water metering system in [32]. However, the researchers in [33] assessed the usefulness of three LoRaWAN frequencies in a water quality monitoring system to determine the use of each frequency. Based on these studies, a system for both water monitoring and leakage detection was necessary. Consequently, the performance of a LoRaWAN within a large housing complex needs to be analyzed to avoid water wastage and to save energy. This study is a simulation of LoRaWAN to be applied in a housing complex to monitor and detect water leakage. This investigation analyzes the usefulness of LoRaWAN’s feasibility, reliability, and scalability for water monitoring and leakage detection in simulated housing complex scenarios.

## 3. Proposed Model

**Figure 2:**
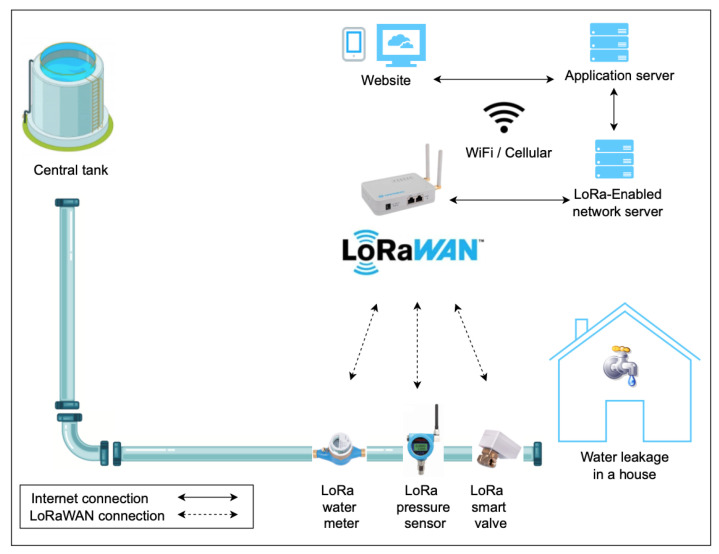
Architecture of LoRaWAN-based water monitoring and leakage detection system.

Monitoring and detecting water leakage is a massive issue in many countries. Although various approaches to this problem are available, the outcomes are still insufficient. Networks for monitoring and detecting water leakage are critical for an efficient water distribution system. Therefore, short-range, high-cost, and high-energy-consumption scenarios require a more specialized network. Consequently, LoRaWAN was used to monitor and detect water leakages in a housing complex. This research proposed a system model using a LoRaWAN via simulation to evaluate the housing complex’s network performance (Figure 2).

The proposed model assumed a central tank for water distribution within the housing complex and pipes. Each house had a water meter, pressure sensor, and smart valve. The water meter measured the amount of water entering each house, and the pressure sensor measured the water pressure in the pipes. The smart valve was closed remotely to stop water leakage. The model detected water leakage using LoRa nodes connected to the Internet through the LoRaWAN gateway. The LoRaWAN used a Wi-Fi or cellular network to access the application and the network servers that enabled it. The application server will send a notification to the homeowner to find a solution to the water leakage issue. Additionally, the smart valve will be closed automatically. In this way, the problem of water leakage is solved by turning off the smart valve and sending a notification to the homeowner through a network with low costs and energy.

The system model was simulated by FloRa on the OMNeT++ platform. OMNeT++ is a C++ simulator toolkit and platform that is flexible and adaptable. It has a component and integrated development environment for visual simulation. The aim of OMNeT++ is the modeling of network technologies and their devices. It is employed in a wide range of domains, including simulators of peer-to-peer, ad-hoc, and mobile networks [34]. FLoRa is a fully accessible simulator package that offers representations for the LoRaWAN network. It facilitated bidirectional communication between the gateway and end devices, and the network servers that constructed the LoRaWAN architecture. The spreading factor, code rate, frequency, transmission power, and bandwidth were only a few features of the LoRa layer that the FLoRa modeled and implemented. FloRa emulates the physical layer by setting all communication parameters and using a simple collision concept that assumes that any two signals traveling across non-orthogonal channels clash when their timestamps match. In place of fully implementing the LoRaWAN standard, the modules handled the Medium Access Control (MAC) protocol [13]. Data were generated from the sensors and stored in the cloud. Each LoRa node in the housing complex consisted of a water meter, water pressure sensor, and smart valve sensor. The LoRaWAN network was used in a housing complex with a square area of 1, 4, 9, 16, and 25 km² with 72, 288, 648, 1152, and 1800 houses, respectively. Therefore, the number of LoRa nodes depended on the number of houses.

**Figure 3:**
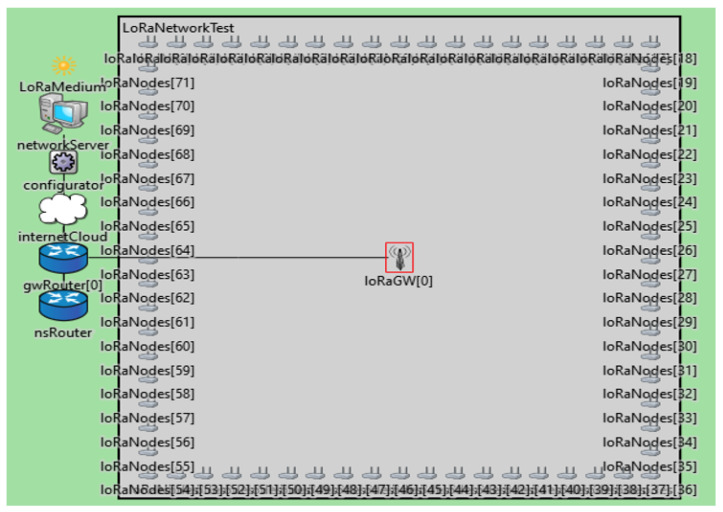
Layout of 72 nodes within a housing complex of 1 km² with a single gateway in OMNeT++.

The simulation ran for seven days. The SF was randomly selected from 7 to 12 at the beginning of the simulation. Then, the Adaptive Rata Rate (ADR), a feature in FloRa, was used to select it during the simulation. The frequency in this system was 868 MHz, which was based on the European Union (EU) standard. Moreover, the simulated system used 4/8 as the coding rate and 125 kHz as a bandwidth based on [13,31,33,35]. The transmission power of each packet was 14 dBm based on [31,35]. Each scenario had 10 iterations. Figure 3 shows the layout of this work as an example of a 1 km² housing complex with a single, central gateway. The gateway of the LoRaWAN is in the center of the layout, surrounded by the red rectangle. The gateway is an intermediate device that forwards data from LoRa nodes to a network server and vice versa. LoRa nodes are located on the edges of the layout, and the number of nodes, in this case, is 72. Each node represents a house and includes the sensors. A network server, a configurator, and a router are located on the left to manage the communication. Hence, this layout is a visual representation inside the OMNeT++ platform of a LoRaWAN network in a housing complex of 1 km².

**Figure 4:**
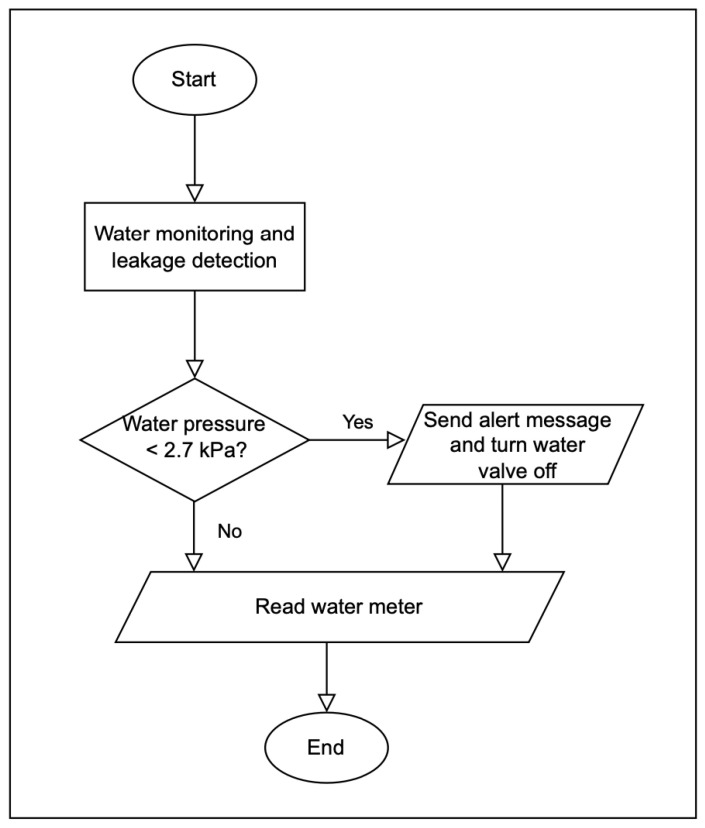
Water monitoring and leakage detection flowchart.

Two scenarios were used to evaluate the proposed model using a LoRaWAN. The first was without any water leakage. The amount of water consumed was measured and sent to the network server every 1800 s (half hour) with 32 bytes in each packet, as in [31]. The second was a water leakage scenario, and this research assumed that it affected 20% of the houses. A message was sent when the pressure fell below 2.7 kPa based on [15]. If the pressure had been less than this value, the node would have sent a message every 300 s to take immediate action. Consequently, the water was controlled and stopped using the smart valve. Figure 4 shows the proposed water monitoring and leakage detection flowchart.

The packet size determined if a house had a water leak. The first scenario used 32 bytes because of the water meter based on [31]. On the other hand, the second scenario used 51 bytes based on [33]. The authors in [33] also used 51 bytes to send more than one value. Consequently, this research did the same to send water meter and pressure values, and smart valve commands in the water leakage scenario. The main parameters in this research are listed in Table 2.

## 4. Results and Discussion

This section contains the results of LoRaWAN water monitoring and leakage detection using FloRa through OMNeT++ in a housing complex. The performance metrics were the packet delivery ratio, energy consumption, throughput, collisions, and traffic distribution over the spreading factor. Simulation scenarios varied with network size and number of nodes.

The first scenario (without water leakage) simulated the LoRaWAN network in 32 bytes for all packets sent every 1800 s. The second (with water leakage) simulated the LoRaWAN network with 32 bytes of packets sent without leakage every 1800 s and 51 bytes of packets sent without leakage every 300 s. Since the network size and number of nodes increased, the packet delivery ratio decreased. On the other hand, energy consumption, collisions, and throughput increased.

### 4.1. Packet Delivery Ratio (PDR)

The packet delivery ratio (PDR) was the proportion of successfully received packets to all the packets transmitted. Measurement software that keeps track of total packets received and transmitted was used to compute the PDR [36]. Equation (1) shows that the sum of successful packets divided by the sum of total sent packets gives the success ratio.

(1) $$\begin{array}{cc} \mathrm{Packet}\mathrm{delivery}\mathrm{ratio} & =\frac{\sum \mathrm{Successful}\mathrm{received}\mathrm{packets}}{\sum \mathrm{Total}\mathrm{sent}\mathrm{packets}} \end{array}$$

**Figure 5:**
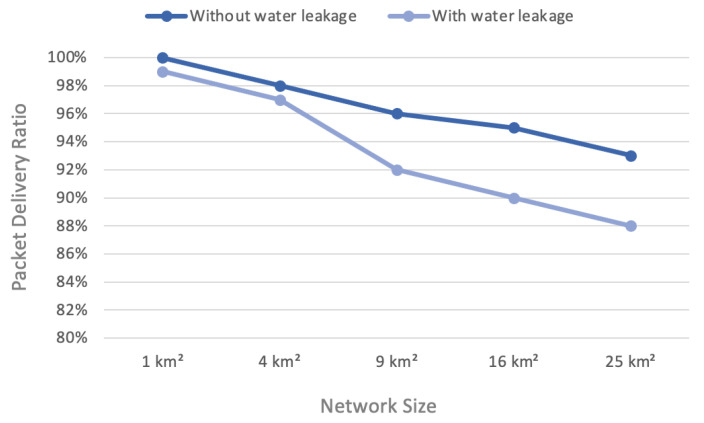
Packet delivery ratio (PDR) versus network size.

Figure 5 shows the success ratio of the network with and without a water leakage scenario for various areas. The success of delivering packages was 100% only in an area of 1 km² without water leakage. On the other hand, the success ratio of the water leakage scenario decreased until it fell to 88% in an area of 25 km².

The PDR slowly decreased as the network size and number of nodes increased, but increasing the network size without increasing the gateways lowered the PDR and thus the delivery ratio to the network server. There appeared to be a difference between the two scenarios. The one without water leakage gave a better result due to the triviality of the payload and the sufficient use interval.

### 4.2. Energy Consumption

Energy consumption is expressed as the power used by all the LoRa nodes in a specific scenario divided by the number of signals received by the network server. Because energy is used for each successful message transmission [13], we used total consumption as a performance metric to assess the energy efficiency of the LoRaWAN in the system model.

In this research, the sum of energy consumed by the nodes divided by the number of nodes gave the network consumption average over a specific time, as in Equation (2).

(2) $$\begin{array}{cc} \mathrm{Average}\mathrm{energy}\mathrm{consumption} & =\frac{\sum \mathrm{Energy}\mathrm{consumption}\mathrm{of}\mathrm{nodes}}{\mathrm{Number}\mathrm{of}\mathrm{nodes}} \end{array}$$

**Figure 6:**
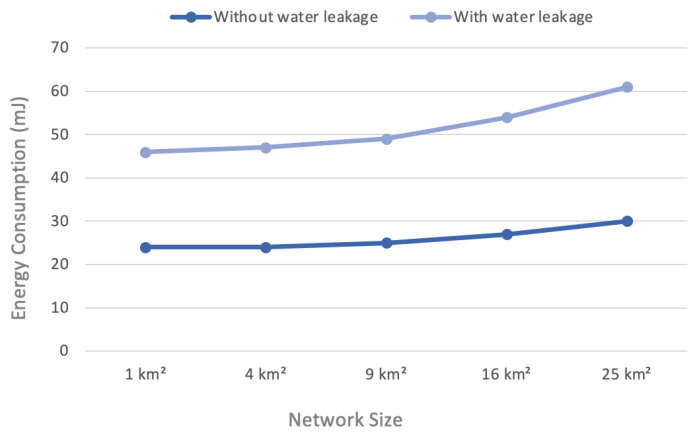
Energy consumption versus network size.

The energy consumption offered acceptable performance for the network requirements, as shown in Figure 6. The average energy consumption without water leakage was better through various scenarios. The average energy consumption with water leakage was 40–70 mJ. The energy consumption without water leakage was 20–40 mJ.

Energy consumption was highly dependent on the size of the network and the number of nodes. When the network size and number of nodes increased, the remote nodes spent more transmission power compared to the near nodes. In the water leakage scenario, energy consumption appeared higher since the network transmitted continuously, thereby consuming more power. Accordingly, the energy consumed in large-area scenarios was greater than for small-areas. Moreover, the energy consumed in the water leakage scenario was greater due to the increased number of transmission times to the network server and the considerable size of the packets.

### 4.3. Average Throughput

Average throughput is the average number of bits per second for network uplinks and downlinks [37]. Equation (3) explains the average throughput used in this research.

(3) $$\begin{array}{cc} \mathrm{Average}\mathrm{throughput} & =\frac{\sum \mathrm{Number}\mathrm{of}\mathrm{uplink}\mathrm{and}\mathrm{downlink}\mathrm{bits}}{\mathrm{Time}(s)} \end{array}$$

**Figure 7:**
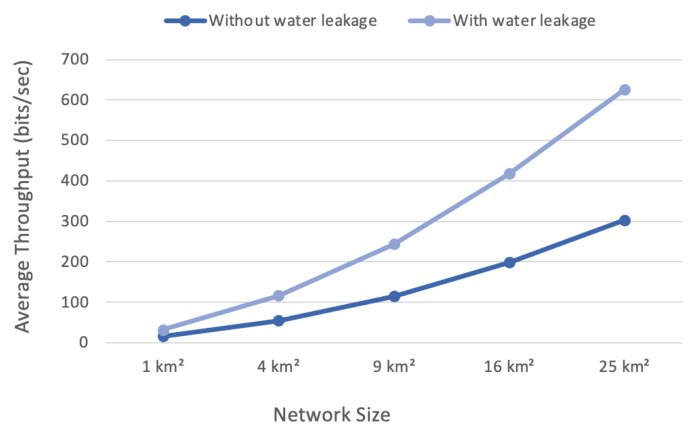
Throughput versus network size.

The average throughput increased when the area expanded, as shown in Figure 7. The larger the network, the higher the throughput. The difference in throughput was not clear in the small network sizes, as in the 1 and 4 km scenarios. In the 25 km² scenario, the difference was meaningful. The scenario without water leakage delivered 300 bits/s, and the one with water leakage produced more than 600 bits/s, which meant that the throughput doubled.

The number of transmitted bits increased as the number of nodes grew. Large areas of the scenarios contained a large number of nodes. Consequently, the throughput increased when the packets increased during a specific period. Furthermore, the throughput was affected by the number of transmissions. The scenario without water leakage had a higher throughput increase because of the greater number of bits in the transmission packets and number of transmissions. When the number of bits transmitted per second increased, the average throughput increased significantly.

### 4.4. Collisions

**Figure 8:**
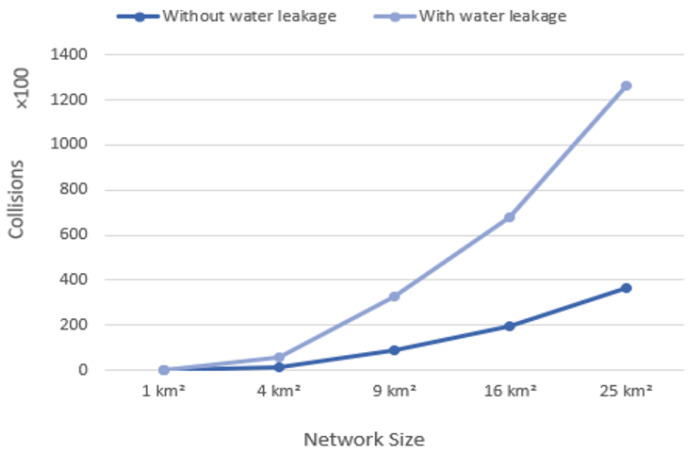
Collisions versus network size.

The gateway records a packet collision if there are at least two concurrent broadcasts on a single frequency channel [38]. Collisions increase as more messages are transmitted over the network. Figure 8 illustrates how collisions rise as the area is extended. Moreover, the scenario without water leakage was superior over areas of 9, 16, and 25 km².

These collisions negatively affected network performance where they increased. The marked difference between these two scenarios was due to the payload size of the packet. Furthermore, short intervals in the scenario with water leakage generated more collisions. The scenario without water leakage using the LoRaWAN provided optimal results compared with the water leakage scenario. This can be explained by the fact that networks with fewer nodes conduct better than those with more. Hence, when fewer collisions happened, performance improved.

### 4.5. Traffic Distribution over Spreading Factor (SF)

The fundamental element of LoRaWAN communication is the spreading factor (SF), which uses an encoding technique to divide a bit into several chips. Each of the SF values, which range from 7 to 12, depends on the quantity of chips [39]. The percentages of received traffic allocated through the spreading factors (SFs) were displayed in various areas with and without water leakage. The simulated system enabled an ADR with random SFs. Consequently, the traffic was selected based on the needs of the network.

**Figure 9:**
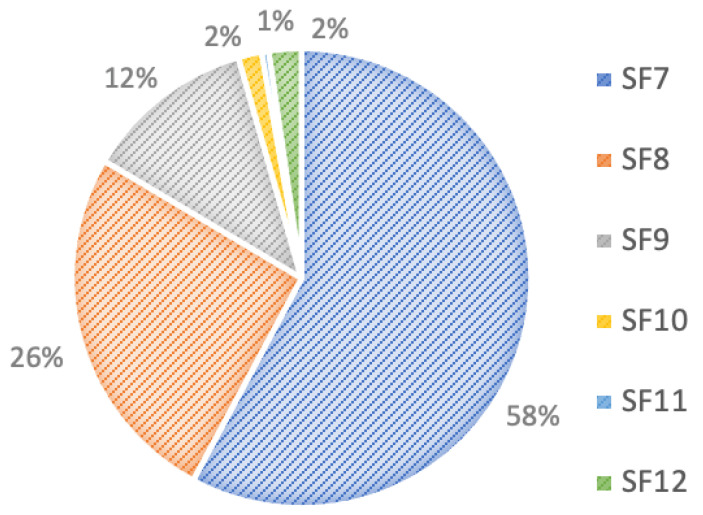
Received traffic for SFs over 25 km² without water leakage.

The received traffic for SFs in the 25 km² area without water leakage is shown in Figure 9. SF7 occupied the largest proportion of the spreading factors, amounting to more than half in this scenario, whereas SF10, SF11, and SF12 together comprised only 5%.

**Figure 10:**
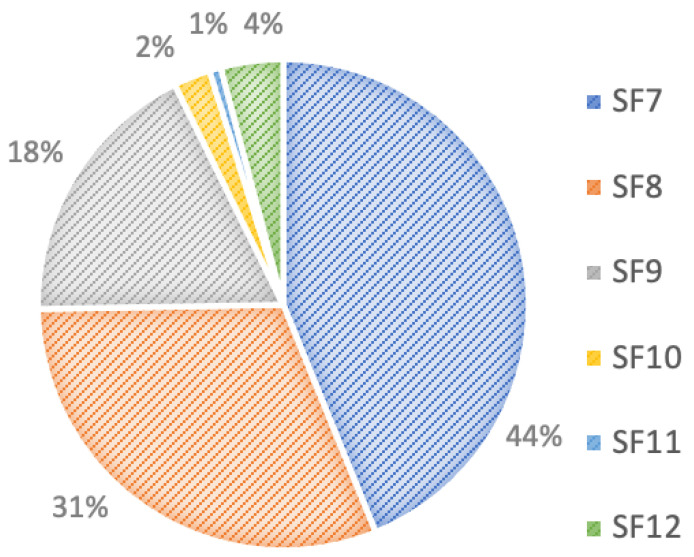
Received traffic for SFs over 25 km² with water leakage.

The received traffic for SFs over 25 km² with water leakage is shown in Figure 10. SF7 occupies the largest proportion of the figure with and without water leakage. Almost all nodes were near the gateway due to the use of SF7. If the node were far away from the gateway, it would use a higher SF. In this experiment, the scenario without water leakage used SF7 more than the scenario with water leakage. The use of SF7 was due to the lightness of the packet and the long interval between packet transmissions.

The results showed satisfactory performance as the LoRaWAN was better than GSM, Wi-Fi, Zigbee, and Bluetooth, which were compared in [19]. Moreover, the performance of the LoRaWAN network was measured by the packet delivery ratio in the water monitoring and leakage detection system, and it outperformed the one in [31]. The worst cases of the LoRaWAN performance, which was 88%, was still better than most results in [31], which used a random SF.

In general, using LoRaWAN instead of GSM is better in terms of cost, scalability, maintenance, and battery life based on [40]. Therefore, the system proposed in this paper is better than the system in [15] due to the use of LoRaWAN.

## 5. Conclusions

In this study, the LoRaWAN’s performance in a water monitoring and leakage detection model in a housing complex was represented. Every house had a LoRa node consisting of a water meter, pressure sensor, and smart valve. We relied on water leakage detection using the low-pressure technique. Next, we measured the LoRaWAN’s performance in two different scenarios. The first scenario was a water monitoring system without leakage and the second scenario was with leakage. Both scenarios showed a high packet delivery ratio. The PDR of the network server reached 100% in the first scenario of 1 km². The water leakage scenario fell to 88% in the 25 km² scenario.

Furthermore, we evaluated LoRaWAN performance for energy consumption, average throughput, collision, and traffic distribution over available SFs. The result showed that a LoRaWAN in an organized housing complex was suitable. The issue of water leakage is solved by stopping the water supply via the smart valve and sending a message to the homeowner through a network with low costs and energy. However, the development of the model in some housing complexes in real life should be carried out more widely. In future work, we will improve this study into reality and develop a web application for water monitoring and leakage detection. Moreover, we will focus on various IoT applications using a LoRaWAN network.

## Figures and Tables

**Table 1:** A comparison of LoRaWAN-based simulated applications for water management systems.

Ref.	Objective	Area Size	Number of Gateways	Number of Nodes	Frequency	Spreading Factor	Location	Simulator
[31]	To determine the number of smart water meters with a single LoRaWAN gateway	177 km${}^{2}$	1	100	868 MHz	7–12	Tunisia	NS3
[32]	To measure LoRaWAN scalability to be used in water-metering grid infrastructure	114.66 km${}^{2}$ rural, 55.66 km${}^{2}$ suburban, 256 km${}^{2}$ urban	2, 4, 14	-	920–923 MHz	7–12	Three cities, Indonesia	Forsk Atoll
[33]	To evaluate the usability of LoRaWAN parameters in a water quality monitoring system	15 km${}^{2}$	1	-	433 MHz, 868 MHz, 915 MHz	7–12	Pune, India	MATLAB (Simulink)

**Table 2:** The main simulation parameters.

Parameters	Value
Simulation time	7d
Spreading factor	7–12
Packet size	32 bytes, 51 bytes
Frequency	868 MHz
Bandwidth	125 KHz
Coding rate	4/8
Transmission power	14 dBm
Simulation area	1, 4, 9, 16, and 25 km²
Number of nodes	72, 288, 648, 1152, 1800
Interval	300 s, 1800 s
Repetition	10 times

## Data Availability

Not applicable.

## References

[1] Muteba F., Djouani K., Olwal T. (2019). A comparative Survey Study on LPWA IoT Technologies: Design, considerations, challenges and solutions. Procedia Comput. Sci..

[2] Mekki K., Bajic E., Chaxel F., Meyer F. (2019). A comparative study of LPWAN technologies for large-scale IoT deployment. ICT Express.

[3] Gambiroza J.C., Mastelic T., Solic P., Cagalj M. (2019). Capacity in LoRaWAN networks: Challenges and opportunities. Proceedings of the 2019 4th International Conference on Smart and Sustainable Technologies (SpliTech).

[4] Torroglosa-Garcia E.M., Calero J.M.A., Bernabe J.B., Skarmeta A. (2020). Enabling roaming across heterogeneous IoT wireless networks: LoRaWAN MEETS 5G. IEEE Access.

[5] Vangelista L., Zanella A., Zorzi M. (2015). Long-range IoT technologies: The dawn of LoRa™. Future Access Enablers of Ubiquitous and Intelligent Infrastructures.

[6] Haxhibeqiri J., De Poorter E., Moerman I., Hoebeke J. (2018). A survey of LoRaWAN for IoT: From technology to application. Sensors.

[7] Khutsoane O., Isong B., Abu-Mahfouz A.M. (2017). IoT devices and applications based on LoRa/LoRaWAN. Proceedings of the IECON 2017-43rd Annual Conference of the IEEE Industrial Electronics Society.

[8] Augustin A., Yi J., Clausen T., Townsley W.M. (2016). A study of LoRa: Long range & low power networks for the IoT. Sensors.

[9] Aldahdouh K., Alouneh S. (2020). Optimizing the energy consumption level in lorawan networks. Proceedings of the 2020 21st International Arab Conference on Information Technology (ACIT).

[10] Zhou Q., Zheng K., Hou L., Xing J., Xu R. (2019). Design and implementation of open LoRa for IoT. IEEE Access.

[11] Lavric A., Popa V. (2017). Internet of things and LoRa™ low-power wide-area networks: A survey. Proceedings of the 2017 International Symposium on Signals, Circuits and Systems (ISSCS).

[12] Noura H., Hatoum T., Salman O., Yaacoub J., Chehab A. (2020). LoRaWAN security survey: Issues, threats and possible mitigation techniques. Internet Things.

[13] Slabicki M., Premsankar G., Di Francesco M. (2018). Adaptive configuration of LoRa networks for dense IoT deployments. Proceedings of the NOMS 2018—2018 IEEE/IFIP Network Operations and Management Symposium.

[14] Liando J.C., Gamage A., Tengourtius A.W., Li M. (2019). Known and unknown facts of LoRa: Experiences from a large-scale measurement study. Acm Trans. Sens. Netw. (TOSN).

[15] Rosli N., Aziz I.A., Jaafar N.S.M. (2018). Home Underground Pipeline Leakage Alert System Based on Water Pressure. Proceedings of the 2018 IEEE Conference on Wireless Sensors (ICWiSe).

[16] Wixted A.J., Kinnaird P., Larijani H., Tait A., Ahmadinia A., Strachan N. (2016). Evaluation of LoRa and LoRaWAN for wireless sensor networks. Proceedings of the 2016 IEEE SENSORS.

[17] Lim J., Lee J., Kim D., Kim J. (2017). Performance analysis of LoRa (Long Range) according to the distances in indoor and outdoor spaces. J. KIISE.

[18] Tsakmakis A., Valkanis A., Beletsioti G., Kantelis K., Nicopolitidis P., Papadimitriou G. (2022). An Adaptive LoRaWAN MAC Protocol for Event Detection Applications. Sensors.

[19] Saravanan K., Anusuya E., Kumar R., Son L.H. (2018). Real-time water quality monitoring using Internet of Things in SCADA. Environ. Monit. Assess..

[20] Olisa S.C., Asiegbu C.N., Olisa J.E., Ekengwu B.O., Shittu A.A., Eze M.C. (2021). Smart two-tank water quality and level detection system via IoT. Heliyon.

[21] Júnior A.C.D.S., Munoz R., Quezada M.D.L. (2021). Á; Neto, A.V.L.; Hassan, M.M.; De Albuquerque, V.H.C. Internet of water things: A remote raw water monitoring and control system. IEEE Access.

[22] Pointl M., Fuchs-Hanusch D. (2021). Assessing the potential of LPWAN communication technologies for near real-time leak detection in water distribution systems. Sensors.

[23] Olatinwo S.O., Joubert T. (2019). Enabling communication networks for water quality monitoring applications: A survey. IEEE Access.

[24] Babazadeh M. (2019). Edge analytics for anomaly detection in water networks by an Arduino101-LoRa based WSN. ISA Trans..

[25] Khutsoane O., Isong B., Gasela N., Abu-Mahfouz A.M. (2019). Watergrid-sense: A lora-based sensor node for industrial iot applications. IEEE Sensors J..

[26] Slany V., Lucansky A., Koudelka P., Marecek J., Krcalova E., Martinek R. (2020). An integrated iot architecture for smart metering using next generation sensor for water management based on lorawan technology: A pilot study. Sensors.

[27] Wang J., Liu Y., Lei Z., Wu K., Zhao X., Feng C., Liu H., Shuai X., Tang Z., Wu L. Smart water lora IoT system. Proceedings of the 2018 International Conference on Communication Engineering and Technology.

[28] Ye Y., Yang Y., Zhu L., Wang J., Rao D. (2021). A lora-based low-power smart water metering system. Proceedings of the 2021 IEEE International Conference on Consumer Electronics and Computer Engineering (ICCECE).

[29] Suryaa K.S., Vigneshwaran S., Sujatha R. (2020). LoRaWAN Based Secured Water Leak Monitoring System. Proceedings of the 2020 IEEE 4th Conference on Information & Communication Technology (CICT).

[30] Sammaneh H., Al-Jabi M. (2019). IoT-enabled adaptive smart water distribution management system. Proceedings of the 2019 International Conference on Promising Electronic Technologies (ICPET).

[31] Lalle Y., Fourati L.C., Fourati M., Barraca J.P. (2020). LoRaWAN network capacity analysis for smart water grid. Proceedings of the 2020 12th International Symposium on Communication Systems, Networks and Digital Signal Processing (CSNDSP).

[32] Bagariang Y., Nashiruddin M.I., Adriansyah N.M. LoRa-based IoT Network Planning for Advanced Metering Infrastructure in Urban, Suburban and Rural Scenario. Proceedings of the 2019 International Seminar on Research of Information Technology and Intelligent Systems (ISRITI).

[33] Alset U., Kulkarni A., Mehta H. (2020). Performance Analysis of Various LoRaWAN Frequencies For Optimal Data Transmission Of Water Quality Parameter Measurement. Proceedings of the 2020 11th International Conference on Computing, Communication and Networking Technologies (ICCCNT).

[34] Wehrle K., Günes M., Gross J. (2010). Modeling and Tools for Network Simulation.

[35] Al Mojamed M. (2022). Smart Mina: LoRaWAN Technology for Smart Fire Detection Application for Hajj Pilgrimage. Comput. Syst. Sci. Eng..

[36] Liang R., Zhao L., Wang P. (2020). Performance evaluations of LoRa wireless communication in building environments. Sensors.

[37] Ali Z., Henna S., Akhunzada A., Raza M., Kim S.W. (2019). Performance evaluation of LoRaWAN for green Internet of Things. IEEE Access.

[38] Markkula J., Mikhaylov K., Haapola J. (2019). Simulating LoRaWAN: On importance of inter spreading factor interference and collision effect. Proceedings of the ICC 2019—2019 IEEE International Conference on Communications (ICC).

[39] Pukrongta N., Kumkhet B. (2019). The relation of LoRaWAN efficiency with energy consumption of sensor node. Proceedings of the 2019 International Conference on Power, Energy and Innovations (ICPEI).

[40] Hattarge S., Kekre A., Kothari A. (2018). LoRaWAN based GPS tracking of city-buses for smart public transport system. Proceedings of the 2018 First International Conference on Secure Cyber Computing and Communication (ICSCCC).

